# Indents

**Published:** 1905-08-15

**Authors:** 


### INDENTS.

☞ **Brief Articles of Technical or Scientific Value will receive notice and comment. Contributions invited.**

**To Make a Good Cotton Holder for a Few Cents.** Get a common vaseline jar with a wide mouth and metal screw cover. Make a round hole in the middle of cover about three-quarters of an inch in diameter with a pointed flat file. Make a spiral spring out of piano wire, place a piece of round cardboard on top of it, place in jar, screw on crown and put in cotton.—*Dental Summary.*

**Shading Facings for Gold Bridges.** In selecting facings for gold crowns or bridges, much care must be exercised to obtain the correct shade. This may be accomplished by the use of a piece of gold or platinum held at the back of the facing, as previously explained by Dr. R. L. Graber, of Peoria, Ills. If the crowns are all on one side of the mouth, each crown should harmonize with the corresponding tooth on the opposite side, providing that the natural teeth have not been discolored. Teeth in one mouth are not all the same color. A good effect is usually produced by backing the lateral with platinum and the cuspid with pure gold then the bicuspid facings with platinum. The central may be backed with the metal which seems best suited for the case, and after a little experience the operator can usually determine at a glance which metal to employ. Try this method and not the results.—I. T. O. R., in *Review.*

**A Broach Holder That can be Sterilized.** The ordinary broach holder is made of celluloid, rubber, bone or wood. The steel chucks were removed from the celluloid handles, and silver tubes made. The chucks were soft-soldered into the end of the tubes, the other end being slightly flattened, to prevent rolling, and left open that water may be shaken from them. These are ideal handles. Cleanly, and the sense of touch is very considerably aided as the silver tubes transmit vibration much more readily than the various animal or vegetable handles. Handles for short broaches are made as follows: A square German silver tube, such as is used for making nuts, for regulating appliances, was sucked full of soft solder. The broach of any kind, is cut the length desired, and the end is caught in the end of a square tube by the soft solder. Then file off an eighth or three-sixteenths of an inch of the tube for a handle. The square handles are superior to the round, and may be grasped by the Bronson pliers and carried to the posterior canals. All these broaches and handles can be placed in the sterilizer and a varied assortment should be kept made up.—Dr. Voyles.

**Tooth Brushes.** Here, too, I have no use for the soft, mushy tooth brush sometimes advocated, except in very rare cases. I want a tooth brush with some backbone to it, with some substance to it. It should be small, but full of good stiff, elastic bristles. Small, to reach every part of the mouth; stiff, to give good stimulating friction to both tooth and gum; straight, not curved, because then best adapted to reach the surfaces on both sides of the arch. It should be used in all directions, vertically and horizontally. The "tooth pick" idea, associated with the dozen or more little bristles as the end of the curved brushes does not appeal to me at all. For daily use to remove particles of food, to brush out the softer debris, it is a necessity. In order to keep it in good condition for this purpose and preserve its vitality longer, one should have two or three always in commission for alternate use. As a polisher, however, the tooth brush is utterly inadequate because even with the use of a good powder it fails entirely to remove the adhesive deposits, which are so prevalent and so mischievous to the teeth, and there are too many places inaccessible to the brush.—Dr. Rohland, in *Review*.

**Peroxide of Hydrogen.** Another comparatively recent remedial agent, and the use of which is attended oftentimes with so much danger, and which so frequently does harm, is peroxide of hydrogen and its analogues. I sincerely believe that peroxide of hydrogen, pyrozone, hydrozone and any of the solutions of $H^2O^2$, are doing more harm in dentistry and in surgery than all the other agents combined. For example, let peroxide of hydrogen be carried down through a tooth canal for the puprose of neutralizing the putrescence of a tooth pulp, should the infectious material be carried through into the tissues surrounding the tooth root, an abscess is almost certain to fo'low. Moreover, if the peroxide of hydrogen is carried through and in such a way as to pass between the periosteum and the bone, it will be almost certain to carry the periosteum away from the bone some distance from the infectious material, and thus carry the infection into parts where it would otherwise never go. Three cases have come under my treatment within the past year of necrosis of half of the lower jaw which was due, I believe, to the injudicious use of peroxide of hydrogen, which was carried into fistulous openings along the border of the jaw, and which dissected up the periosteum far back, carrying infection where it resulted in destruction of the bone.—T. W. Brophy, in *Review*.

**Sodium Chloride for Pyorrhea.** Dr. E. H. Allen, of Freeport, Ills., gives this history of two cases of pyorrhea. A patient applied to me for other dental services who had lost a large number of his teeth with the real pyorrhea; he had been for treatment to a number of dentists. I have known him for a great many years, and about three years ago he came to me. He told me how many teeth he had lost. He told me that he had just pulled them out himself, but that he had cured all those remaining. He said that he had tried everything that he ever heard of, but that he cured the disease with the application of common salt, and he did, too. The man is nearly seventy years old, and he has no pyorrhea about his mouth now. His gums are solid and healthy, and the recession has stopped. His teeth are so firm that I had no hesitation in making a bridge and attaching it to those teeth. He said that he used to work the salt down into the sockets with a tooth pick. He did that several times each day, and when he brushed his teeth he used salt. I induced a lady teacher to try this method. Her pyorrhea was not so general as the other case, but she could squeeze pus out of the sockets, particularly of the superior incisors, at any time. She used the salt treatment, and I saw her about three months later, when the condition had almost entirely disappeared. There were only one or two places where I found any pus whatever.—*Review.*

**Retaining Loose Teeth.** The appliance consists of what may be styled two yokes held together by means of bolts or screws. These yokes fit accurately the cervical border of the labial and lingual surfaces of the loose teeth and those intended as supports at either end, and are held in place by the bolts which pass through the interproximal spaces. The method of procedure for making such an appliance is as follows: An accurate impression is obtained of the labial and lingual surfaces of the teeth involved from which a metal die is made. On this die is swaged or burnished a narrow strip of pure gold, thirty gauge. This strip is then cut to shape and tried in the mouth and burnished so as to fit the cervical border of the teeth accurately. The yokes should now be strengthened with 22-karat solder. Drill holes the exact gauge of the bolts through the labial yoke at the approximal spaces. Place the yokes in position and with a sharp instrument mark on the lingual yoke where the corresponding holes are to be drilled. The bolts are made of platinum and iridium wire, with cone-shaped head and nuts, which are to be countersunk in the yokes. Where it is necessary to hold an elongating tooth or to prevent the yokes slipping on the roots, a half cap may be swaged to fit the lingual surface of the tooth and soldered to the lingual yoke. This cap is cemented to the tooth, the yokes bolted firmly in place, and the heads and nuts ground and polished smooth. This appliance may be used in every case where the interproximal space is sufficiently wide to permit the passage of a bolt.

The small nut furnished with the Bryant crown and bridge repair system will be found very useful in connection with this method.—E. Darwin Reed, *Dental Brief.*

**The Necessity for a Vacation.** There is no class of workers in this modern human hive upon whom the burden of continued effort falls more heavily than upon dentists. The environment and the nature of the work is such that the monotony and nervous tension become almost unbearable if followed too long without a break. The physician and lawyer have diversity with their work; the mechanic at the bench labors upon inert material, free from the tingling resentment from human nerves. And the dentist is obliged continuously to listen to a recital of all the petty ills from the ancient "gum-boil" to the modern "automobile-face." Small wonder that dentists break down early unless they get away from the office at intervals and forget at the time that they are dentists. The man makes a mistake and limits his efficiency who remains year in and out at the chair and becomes steeped in the associations of so narrow an environment. Better economize in some other way than to attempt to save money by staying in the office the year round and becoming stale and jaded.

The men who accomplish most are those who renew their energy at intervals by a complete change of scene and by devoting their attention occasionally to an entirely different line of activities from those of their routine work. Get out in the woods or on the water and take a breath of nature. After all, when one comes to think of the artificiality of modern life, there is nothing so refreshing and life-renewing as getting back again to first principles and living close to nature. Put on old clothes, live in the open air and rough it generally at least once a year. The sum total of accomplishment will ultimately be far greater than if all the time is spent in a steady grind, and the diversion will give a spice to life which a dentist sadly needs.—Editorial in *Dental Review*.

**Sterilizing Instrnments.** The cleanly practice of dentistry, as I conceive it, is as follows: A clean napkin on the head rest, and an aseptic paper cover on the bracket table. A sterilized mirror and cotton pliers in the warm water heater. The patient is seated, the chair adjusted, and a freshly laundried table napkin pinned to the shoulders of the garment.

In sight of the patient the hands should be well washed and carefully dried. Then take a clean towel from the cabinet, wipe the miror and pliers carefully, place them on the bracket table, and hang the towel on the back of the chair. If the saliva tube is to be used, take out the one in the cuspidor and replace with a clean one. Then proceed with the work in hand, using sterilized napkin and cotton rolls. When the operation is completed, while the patient is getting out of the chair, lift off the bracket table cover with all instruments used and carry to the washstand. The instruments should all be placed in the tray of the sterilizer and held under the faucet for a thorough washing. Then place them in the sterilizer and turn the gas on full. All this can be done while the patient is getting his or her clothes straightened out, and unloading a few farewell remarks. Usually the water will be in full boil before the patient is gotten out of the room. The next patient is brought into the operating room, and a clean cover is placed. Another mirror and pliers are warmed, and the patient seated. If you have an assistant she should remove the instruments from the sterilizer, dry, and replace them in the cabinet. If you work alone, stop and do it yourself. It takes not more than two minutes, and you can afford to spare the time for the impression it makes on the patient.

This is the practice in my office, but to reach it has cost a little money and a considerable amount of study and labor.—Dr. Voyles, in *Era*.

**Why Round-faced Pluggers Should be Employed.**

The *spreading* properties of gold are so well known that it seems scarcely worth while to dwell upon them, yet there are many dentists who fail to take advantage of the well-known characteristic in operative procedures. A mechanic understands the value of a convex face on one of his hammer heads when riveting a bolt or spreading a metal plate. The dentist in his laboratory knows how to take advantage of the spreading property of gold in swaging a plate around a remaining tooth, or over the heel of the process, or, if he has made a crown band too small, it is readily stretched by laying over the convex surface of a projection from the anvil and tapping with a flat-faced hammer, thereby spreading portions of the mass. The gold beater depends upon the convexity of his hammer as he beats the gold leaf to spread it in the vellum cutch or molds from an inch square to the usual four-inch square size. Indeed, the malleability of gold seems to be well understood by our profession in every department of constructive work, except operative procedures at the chair. If search be made through the pluggers of the supply houses, but very few will be found whose impacting points are other than flat, whatever the form of the plugger end may be, and fewer still will be found, in proportion to the number kept, in the individual dental equipment; yet a mass of gold melted and run into a cylindrical form and placed in a circular cavity with parallel walls, not too deep, and just large enough to take the cylinder—allowing it to slip in and out readily—can be so "riveted" or spread in the cavity with a convexed faced plugger that it will be made to fit the cavity and hermetically seal the orifice. How much better this can be done by spreading the gold piece by piece as inserted, spreading the mass laterally, causing it to hug tightly the cavity wall from the base or seat to the surface. I feel sure that practitioners generally have not understood the case with which moisture-tight fillings can be made by taking advantage of this characteristic of gold, and that a flat-faced plugger will not spread the mass.—Dr. D. M. Cattell, *Review.*

**Pyorrhea Treatment.** First, insisting upon the thorough co-operation and support of the patient. I will not take a case unless the patient will carry out my instructions absolutely. I cannot afford to do it, and neither can you. Second, success depend upon clean teeth. I want to emphasize that because the teeth must be clean in all varieties of the disease. There are many of these cases that probabiy would be termed purely phagedenic pericementitis where there are no perceptible deposits; but after these years of experience I am prepared to say that you cannot get results until you have scraped and polished the root, and in many cases scraped the alveolar socket. I think that my fingers are pretty well trained to the handling of scalers, and I know that it is not possible for me, except by local anesthesia, to always accomplish this result painlessly.

In all inaccessible positions I invariably pack the pocket first. I syringe it out and clean it antiseptically and pack it in such a way as to lift out temporarily the loose tissue, so that on future occasions I can see the surface that was hidden from be at the beginning. In those bad, inaccessible places I don't rely altogether on the sense of touch to know that the tooth has been cleaned and polished. I may be forcing away tissue which is already too far away from the tooth, but I maintain that if this can be done without seriously injuring it, and get the tooth surgically clean as well as the pocket, and then force back the tissue, I have brought healthy tissue down upon healthy surface, and I usually get good results. I pack it away with gauze cut in such shape as to be most convenient for the desired position, and usually for the last five years I moisten it in a 25 percent solution of pheno-sulphonic acid. This is not strong enough to be corrosive, but it is styptic and will control the bleeding. It also has a softening influence upon the calcific deposits. I have proved to the satisfaction of a great many who have watched it that it will dissolve deposits, and certainly in many cases where it was difficult to even scratch the deposits, so hard were they, before applying this treatment, afterward they could be removed very easily. Then if the case be one of extensive pocket, and by that I mean enveloping, perhaps, half the root of a tooth, I may keep it packed, and make it heal from the bottom as a surgical wound. Of course, if the peridental membrane has entirely lost its attachment it will never be re-established, and the best we can obtain is a mechanical union. I sometimes, in these very severe cases, keep the pocket packed until it heals from the bottom —in other words not to allow the gum to draw tight at its margin, leaving a large cavity above which is going to be filled with infective material, seeping in and retained there because of the hugging of the gum at its margin. I sometimes find it necessary to make an opening through the gum high up rather than to stretch it at its margin, and clean the place through that.

When all has been said it yet remains true that you never can succeed in treating these cases until you educate yourself to appreciate the different pathological conditions that are manifested in the different cases, and train your fingers to clean the teeth.—E. MaWhinney, in *Review*.

**Millions in Gold Fillings.** Of the wealth of this country there is a certain part that has never entered into the reports of the census or fiscal departments of the Government. There is, if the estimates of men who should know are to be taken as authoritative, over $15,000,000 personal wealth in the shape of gold, of which the Government is entirely ignorant, or, if not ignorant, has decided not to mention it in its financial reports. Each year there is over

$2,000,000 worth of gold that disappears, says the *Boston Post,* that is lost to the moneys of the world, and yet it is not lost. More than $2,000,000 worth of gold is used anually in caring for the mouths of the people of the United States.

This $2,000,000 is practically wealth that is fixed in and about the teeth of patients; it ceases to exist as a precious metal of importance in the world of finance and is only a filling in a tooth, lost forever as an article for which men have fought and died and murdered and perjured and sold themselves since the world began.

These figures seem vast, when the small amount used in making a filling is considered, but they are undoubtedly correct.

The man in charge of the gold department of the largest dental supply house in America arrived at these figures after extensive research and calculations, and his estimate is verified by others in a position to know. Two million dollars taken out of its stock of gold is the price this country pays annually for dental gold.

Ten years ago it is estimated that only 25 percent of the people of this country ever went to a dentist, except to have a tooth pulled.

Five years ago the percentage was 33⅓. Now it is declared that nearly 50 percent of the population pay visits to the dentist's chair for some other reason than that of having an aching tooth extracted.

This increase in the number of dental patients has resulted in a consequent increase in the amount of gold used for this purpose. Practically all of the 50 percent who are numbered as dentist's patients have some gold in their teeth.

Thus ten years ago there was only about $1,000,000 worth of gold used annually for filling purposes as against the $2,000,000 of to-day,

Using the rate of increase of the last ten years as a basis, the average yearly consumption of the precious metal in this manner has been about $1,500,000 a year.

This would make about $15,000,000 worth of gold which has been placed into teeth since 1895. For this amount could be built three of the best battle-ships in the world, the President's salary could be paid for thirty years, every voter who went to the polls last fall could be paid in the neighborhood of $2 for his trouble.

For this amount 10,000 persons could acquire a dental education, providing that they worked during vacations and saved during these months enough to pay for their instruments, and also to lay aside an amount to be called an emergency fund, to refurnish instruments, etc., which are borrowed (?) and become so attached to their new surroundings that they never return.—H. B. Millen, in *Stomatologist.*

**Tempering Steel.** Tempering steel is a process which gives it any degree of hardness. Sudden cooling from a high heat hardens and slow cooling softens this metal. The hardening liquid is usually water or oil of mercury may be used. Good tool steel highly heated and cooled in mercury will become hard enough to cut nearly all known substances.

If a small bar of tool steel six or eight inches long is nicked deeply at intervals of three-fourths of an inch or an inch and heated so the extremity shall be white hot and the heat gradually lessening until the last piece is below red heat, the different sections will show the colors as follows:

1. White hot and scintillating.
2. White hot.
3. Bright yellow.
4. Low yellow or orange.
5. Bright red.
6. Dull red.
7. Low red.
8. Black.

Quenching in cold water the pieces will be found as follows if tried with a file.

1. Too hard to cut. Will cut glass.

2. Almost as hard and brittle as No. 1.

3, 4. Very hard.

5, 6. Hard enough for ordinary tools.

7. Hard enough for tap steel.

8. Unhardened.

Texture and color:

1. Coarse, yellowish and very lustrous.

2. Coarse but not quite so yellow.

3. Finer than No. 1 and 2, with firey lustre.

4. Like No. 3, but not quite so coarse, yet coarser than No. 8.

5. Same grain as No. 8, having a firey luster.

6. Finer than No. 8, hard and strong.

7. Refined and hard on corners and edges, center tough.

8. Original grain.

From this the following rules may be established:

1. Any difference in temperature which may be shown by the color will cause a corresponding difference in the grain.

2. Any temperature which will open the grain so the hardened piece will be coarser than the original bar will make brittle and liable to crack.

3. Temperature high enough to cause a piece to harden throughout but not enough to open the grain will cause the piece to refine and carry a good cutting edge.

4. Temperature which will harden and refine the edges but not clear through is just right for taps and complicated cutters of any shape.

To restore steel to original texture heat for from ten to thirty minutes to a good red heat and cool slowly. A restored piece is never quite so good as the original.

Tempering must be done carefully. Cooling should be rapid and complete. The cooling bath should be large.

The lowest heat at which steel will harden is the best.

Drawing the temper is heating the hardened steel until the scales of oxid on its surface assume a certain color, which is constant or nearly so for the same quality of steel.

**Office Visitors.** They were a little miss of about twelve years old and her English maid with a polite note from the mother asking that we be especially careful and tender in the treatment of her little girl, who had tooth-ache. It seems to be the custom of the wealthy from the North who have been abroad, and the most of them have, and those who havn't are catching on, to turn over the care of their children to nurses, maids, governess and tutors, who are held responsible for their welfare, in imitation of the nobility of Europe. This custom relieves the dear parents of much care and worry, and gives them more time to their seemingly one object in life, which is to get as much pleasure out of it as possible.

A "good morning" addressed to the little lady received a short similar reply. An inquiry as to what and where was the trouble was received with silence but with a turn of the head and a look to the maid. "Oh, she's 'ad a beastly ache in that lower tooth the livelong night, so that she didn't sleep a wink and nor I ither. Yo'll av'e to pull it shure." We made an examination and found a six-year old molar with a cavity on the distal side, with hypertrophied gum, even with the top of the tooth and extending into the cavity, so as almost to prevent access to the exposed pulp, with severe bleeding. A most difficult case to treat. "We will have to devitalize the nerve," we said, "and then fill the roots, etc." "Kill the nerve you mean? Aw' that will never do, that will turn the tooth black, it always does. Er mother would blame me severely if I consented to that. See my hown front teeth 'ow it was made black by killing the nerve?" It took a good long time in argument and explaining, to gain the consent and confidence of the stubborn English spinster for me to get to work on the case. "Why kant you put something into the tooth to relieve the pain and then stop it with a soft substance that can be taken out heasy if it gets to haking again?" Again we argued that such work was only temporary and would certainly result in agravated trouble. We mentioned the use of cocaine, but met with a serious remonstrance. "No sir, kant consent to the use of it. We e'rd of a dentist seriously injuring a woman with it just before we left New York." We found we had a proposition before us, a nervous, suffering child, and an ill informed but conscientious maid. To get the gum out of the way so that the arsenic could be effectually applied and without the use of anything to anesthetize it, made us decide to risk the damaging application of the medicament alone, gently forcing it between the bottom of gum and the point of exposure, depending on the corroding action of the drug to do away with the growth. They were dismissed, but in two hours time were back in the office, the little patient in tears with pain. We recommended an internal remedy. "Not for the world," rejoined the maid, "er mother would never forgive me for allowing 'er to be drugged." Removing the temporary stopping, we found that its pressure had given us enough space to reach the point of exposure, so we made an application of a soothing nature and again applied arsenic, placing over it a cavity cap disk to prevent pressure. This had the desired effect, for they did not return until next morning. The gum now was thoroughly inflamed, and our efforts were directed toward its cure. For ten days, in spite of the regular appointments, morning and afternoon, outside of business hours, that persistent maid brought the child to our office. "Just to 'ave you take a bit of a look at it, don't you know, to keep it doing right." Well, at last we were able to cure the pains, fill the tooth and dismiss the patient and send in a good size bill. To our surprise it did not get to an agent's hands and take a month or more for payment, for these folks "are not to be bothered with money matters." The mother wrote us a kind letter of appreciation and enclosed a check covering double the amount of the bill.—Editorial, *Dental Hints.*

